# A circadian rhythm-related gene signature associated with tumor immunity, cisplatin efficacy, and prognosis in bladder cancer

**DOI:** 10.18632/aging.203733

**Published:** 2021-12-03

**Authors:** Ranran Zhou, Xinyu Chen, Jingjing Liang, Qi Chen, Hu Tian, Cheng Yang, Cundong Liu

**Affiliations:** 1 Department of Urology, The Third Affiliated Hospital of Southern Medical University, Guangzhou, China; 2 The Third School of Clinical Medicine, Southern Medical University, Guangzhou, China; 3 Department of Cardiology, Shunde Hospital of Southern Medical University, Foshan, China

**Keywords:** circadian rhythm, bladder cancer, prognosis, cisplatin, immunity

## Abstract

Circadian dysregulation involves malignant tumor initiation and progression, but the understanding of circadian rhythm’s roles in bladder cancer (BCa) remains insufficient. The circadian rhythm-related genes were collected and clustered based on the Cancer Genome Atlas (TCGA), and the clustering was significantly associated with the prognosis and risk clinicopathological features. Through genomic difference analysis and gene pairing, a circadian rhythm-related signature was successfully established. Kaplan-Meier survival analysis and time-dependent receiver operating curves displayed that the prognosis model was a reliable prognosis biomarker both in the training cohort (n = 396, P = 2.687e-10) and external validation cohort (n = 224, P = 1.45e-02). The patients with high risk have high immune infiltration and high expression of immune checkpoint genes, which partly account for the poor prognosis. TIDE algorithm and the validation in IMvigor210 cohort indicated that the risk signature was a promising marker for the immunotherapeutic response. The risk model could also predict the therapeutic response of cisplatin, which was validated in the Genomics of Drug Sensitivity in Cancer database (P = 0.0049), TCGA (P = 0.038), and T24 BCa cells treated with cisplatin. The functional enrichment showed the risk model was significantly correlated with some malignant phenotypes, such as angiogenesis, epithelial-mesenchymal transition, and KRAS signaling pathway. Totally, we proposed a novel circadian rhythm-related signature for prognosis evaluation, which also helped to predict the immune infiltration and cisplatin sensitivity in BCa.

## INTRODUCTION

Bladder cancer (BCa), one of the leading causes of human death worldwide, carries high morbidity and mortality. The inconspicuous early symptoms make a large number of patients have local metastasis when they are clinically diagnosed [1]. Despite the considerable progress made in medical therapy, such as cisplatin-based neoadjuvant chemotherapy and immune checkpoint inhibitors (ICIs), the 5-year survival rate of muscle-invasive bladder cancer (MIBC) is dismal 5%-20% [2]. Therefore, precise and reliable prognosis prediction is always a hot topic in the field of BCa.

With the development of gene sequencing and big-data analysis, some gene-based models associated with BCa prognosis have been proposed [3, 4]. The established models were always based on some biological functions or processes, such as ferroptosis [5], hypoxia [6], and smoking [7], providing useful clinical tools and cut-in points to investigate the mechanisms. Nevertheless, seeking more accurate predictions remains essential and meaningful.

Circadian rhythm is a phenomenon in the life activities of the body, such as physiology, biochemistry and behaviour, which are periodically driven by the clock genes and clock-control genes, and the period is approximately 24 hours [8]. The circadian rhythm plays an essential role in maintaining homeostasis. Epidemiological studies have found that circadian disorders caused by shift work may increase the risk of cancer [9]. The relationships between malignant tumors and circadian rhythm receive more and more attention [10, 11]. However, few studies focus on circadian rhythm functions in BCa for the moment, and further researches are urgently demanded.

Here, we identified circadian rhythm as a prognostic factor for BCa prognosis via unsupervised clustering and screened multiple biomarkers to construct a circadian rhythm-related signature to evaluate overall survival (OS). To achieve a widespread utility, we adopted a gene-pair strategy for the model establishment, and there is no need for a definite gene expression value [12]. The predictive value of the established model was validated in different independent cohorts. Besides, the associations of the risk signature with tumor immune infiltration and immunotherapeutic response were explored. The predictive value to cisplatin effectiveness was also detected through multi-database analyses and vitro experiments.

## RESULTS

### Circadian rhythm was associated with prognosis in BCa

**Figure 1:**
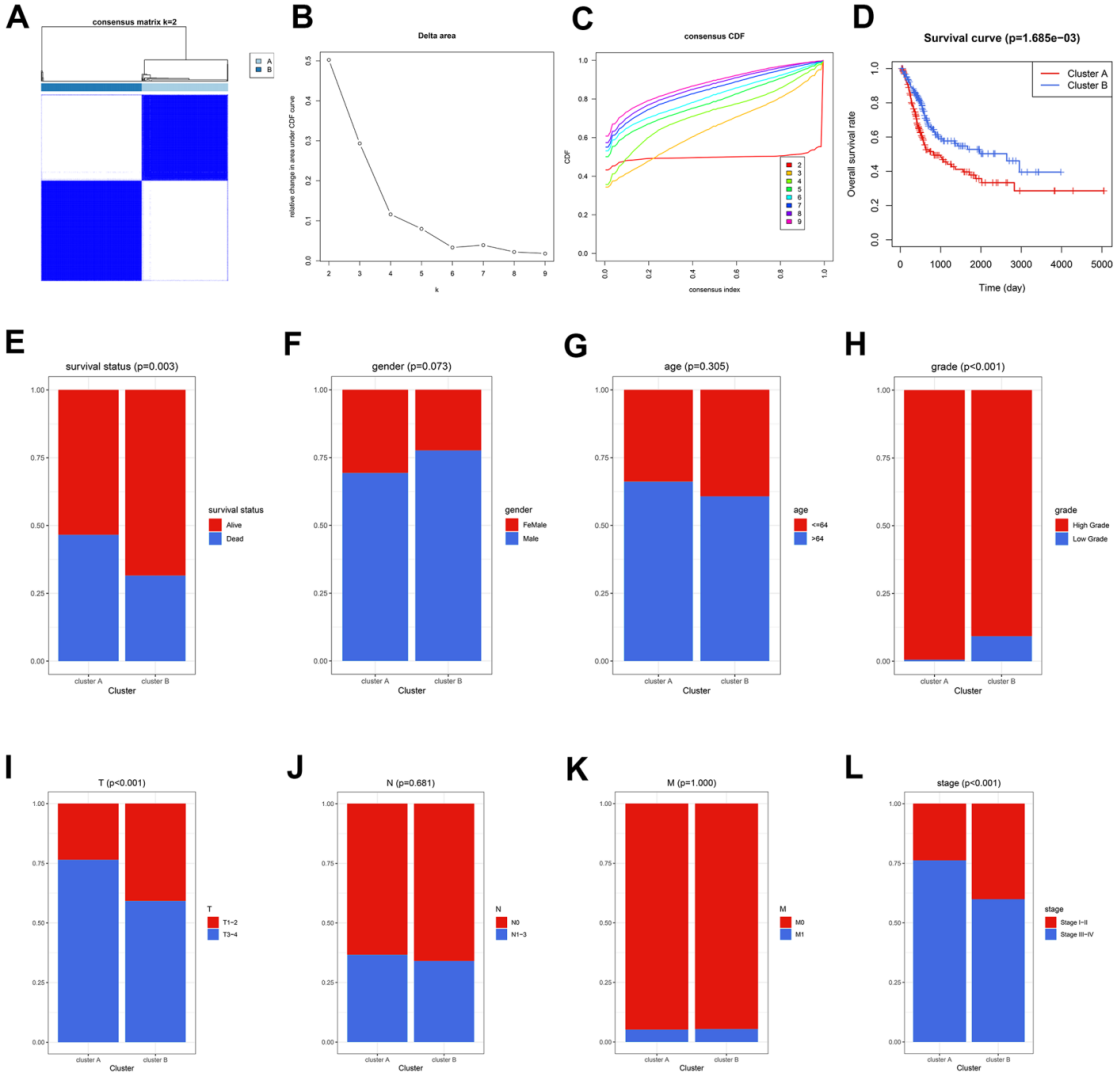
**The circadian rhythm was significantly associated with BCa prognosis.** (**A**–**C**) The BCa patients were divided into two circadian subgroups via unsupervised clustering. (**D**) The Kaplan-Meier analysis displayed that the cases in Cluster A exhibited a worse prognosis. (**E**) More deaths were observed in Cluster A. (**F**–**L**) The association of circadian clustering with gender (**F**), age (**G**), grade (**H**), pathological T stages (**I**), pathological N stages (**J**), M stages (**K**), and tumor stages (**L**). BCa, bladder cancer.

The circadian rhythm-related genes were retrieved from the Molecular Signatures Database (MSigDB, https://www.gsea-msigdb.org/gsea/msigdb/), as displayed in Supplementary Table 1. A sum of 290 circadian-related genes was extracted after excluding the overlapped genes. Accordingly, the Cancer Genome Atlas (TCGA) cases were clustered into two subgroups, containing Cluster A and B, via unsupervised clustering (Figure 1A–1C and Supplementary Table 2). The patients in Cluster A exhibited worse OS compared with those in Cluster B (P < 0.01, Figure 1D), and more deaths were observed in Cluster A (P < 0.01, Figure 1E). Besides, the circadian clustering was also significantly associated with the risk clinicopathological features, such as tumor grade (P < 0.001, Figure 1H), pathological T stages (P < 0.001, Figure 1I), and tumor stages (P < 0.001, Figure 1L), while gender (Figure 1F), age (Figure 1G), pathological N stages (Figure 1J), and M stages (Figure 1K) showed statistical non-significance. These analyses suggested that the critical role circadian rhythm played in BCa might be underestimated, and further exploration was demanded given the previous poor reports.

### Development of a circadian rhythm-related gene signature

**Figure 2:**
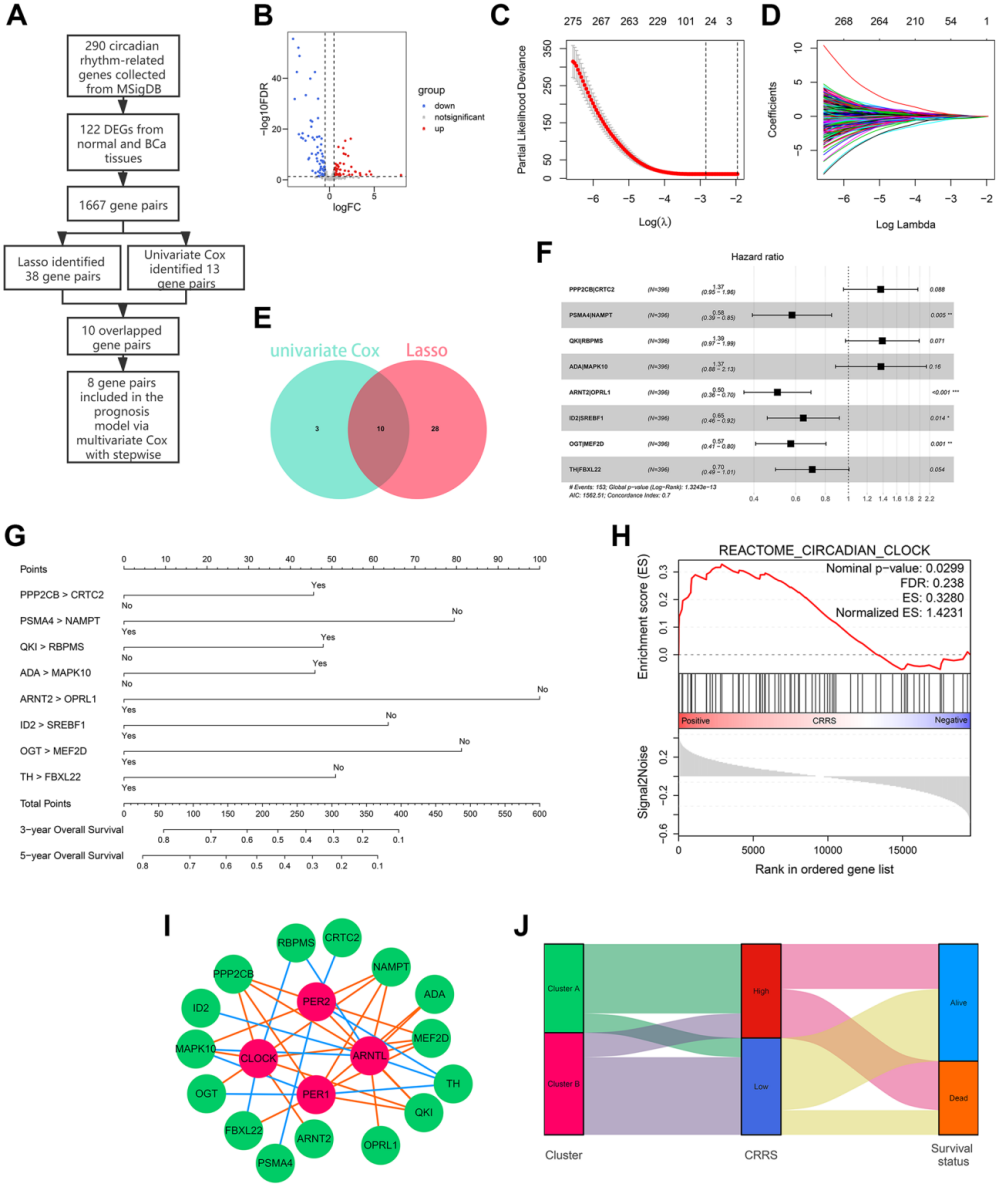
**Development of a circadian rhythm-related signature.** (**A**) The process of the prognostic model construction. (**B**) The volcano plot displaying 122 of 290 circadian rhythm-related genes were differentially expressed between adjacent normal and BCa tissues. (**C**, **D**) Lasso regression identified 38 gene pairs correlated with BCa prognosis. The lines with different colors represented different variables. (**E**) 10 gene pairs were con-determined via univariate Cox regression and Lasso algorithm. (**F**, **G**) The forest plot (**F**) and the nomogram (**G**) of the established model. (**H**) The circadian clock pathway was significantly up-regulated in patients with high CRRS. (**I**) The correlation between the genes in CRRS and known circadian transcription factors. The green bubbles and red bubbles represented the CRRS genes and transcription factors, respectively. The red lines and blue lines represented the positive and negative correlations, respectively. (**J**) The Sankey plot indicated the association between circadian clustering, CRRS stratification, and survival status. BCa, bladder cancer; CRRS, circadian rhythm-related score.

The flow chart of the risk model construction is displayed in Figure 2A. The circadian genes with significant expression differences were chosen for further analysis, and 122 differentially-expressed genes (DEGs) between normal and BCa tissues were screened (Supplementary Table 3 and Figure 2B). Afterwards, 122 genes were cyclically paired, and 1667 gene pairs were established based on the gene-pair strategy [13], among which 38 pairs were associated with OS employing Lasso regression (Figure 2C, 2D). Univariate Cox analysis indicated 10 of the 38 genes pairs carried significant prognostic value with P < 0.001 filterings (Supplementary Table 4 and Figure 2E), and 8 of 10 pairs were ultimately included in the prognostic model through multivariate Cox analysis with stepwise (Supplementary Table 5). To help clinicians better understand the risk model, we drew a forest plot (Figure 2F) and a nomogram (Figure 2G). Here, the risk evaluated by the established model of each case was defined as the circadian rhythm-related score (CRRS). The CRRS was calculated as follows: 0.314*(PPP2CB|CRTC2) – 0.547*(PSMA4|NAMPT) + 0.330*(QKI|RBPMS) + 0.316*(ADA|MAPK10) – 0.688*(ARNT2|OPRL1) – 0.437*(ID2|SREBF1) – 0.559*(OGT|MEF2D) – 0.350*(TH|FBXL22), where (gene A| gene B) represented a gene pair. The value of this pair would be considered as 1 if the expression of gene A is higher than that of gene B; otherwise, it would be defined as 0. Subsequently, we conducted the functional enrichment of the high-CRRS patients via GSEA software (version. 4.1.0) and the reference gene sets associated with circadian rhythm were downloaded from MSigDB (Supplementary Table 1). Gene Set Enrichment Analysis (GSEA) showed circadian clock pathway was significantly enriched in the cases with high CRRS (Nominal P < 0.05, Figure 2H). The expression association between the 16 genes, which comprised the CRRS, and four known circadian transcription factors, including CLOCK, ARNTL, PER1, and PER2, were displayed in Supplementary Table 6 and Figure 2I, and most of the 16 genes exhibited significant correlation. The Sankey diagram showed the distribution of the patients in circadian clustering, CRRS estimation, and survival status (Figure 2J).

### Validation of CRRS

**Figure 3:**
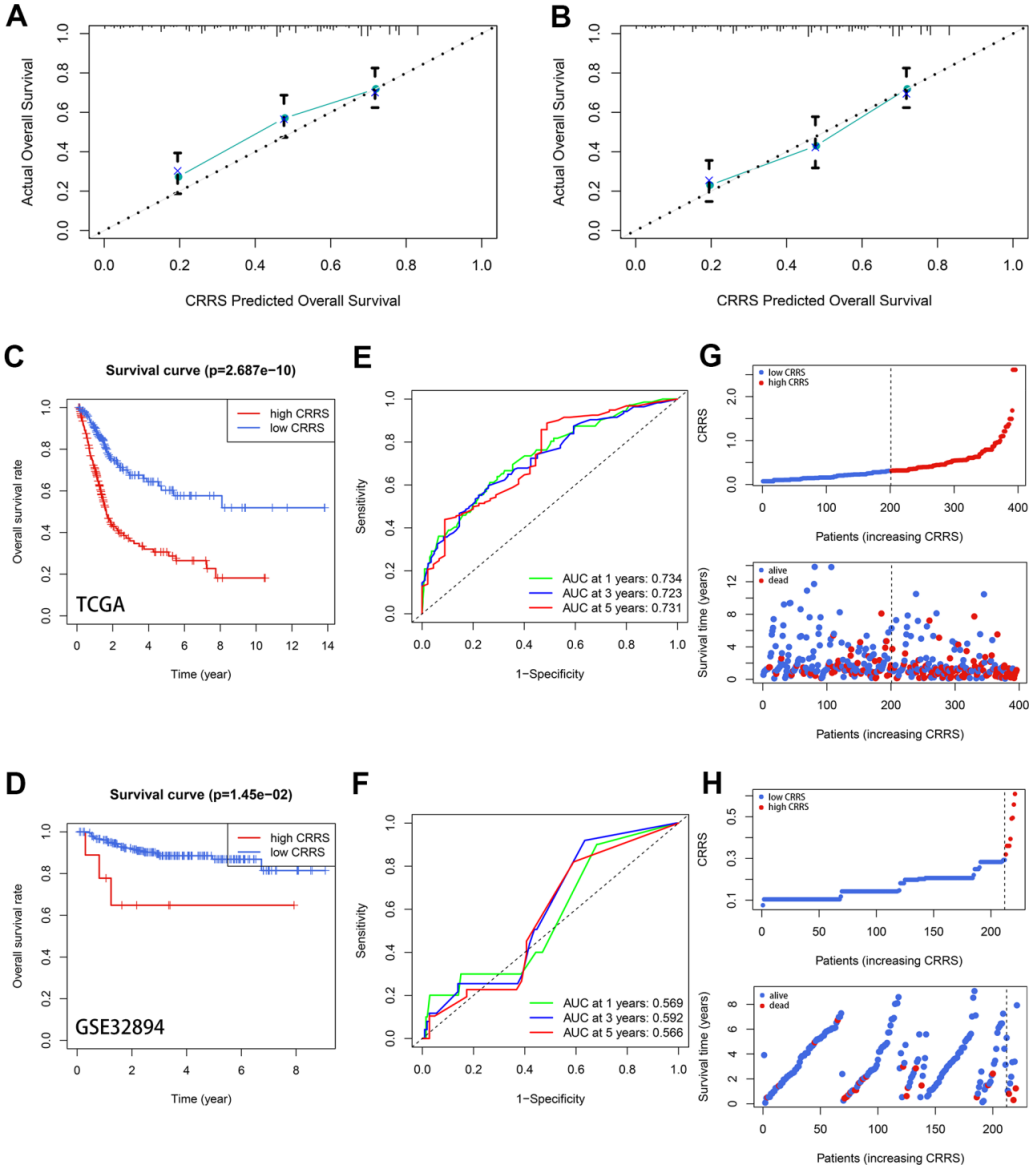
**Validation of the prognostic value of CRRS.** (**A**, **B**) The 3- (**A**) and 5-year (**B**) calibration plots. (**C**, **D**) The Kaplan-Meier survival analysis with a log-rank test in TCGA-BLCA cohort (**C**) and GSE32894 cohort (**D**). (**E**, **F**) The time-dependent ROC curve in TCGA-BLCA cases (**E**) and GSE32894 cases (**F**). (**G**, **H**) The distribution of the CRRS (up) and survival statuses (down) in the TCGA-BLCA cohort (**G**) and GSE32894 cohort (**H**). CRRS, circadian rhythm-related score; TCGA, the Cancer Genome Atlas; ROC, receiver operating curve.

Diverse methods were conducted to validate the robustness of the CRRS in different independent cohorts, including TCGA-BLCA and GSE32894. The baseline clinical traits of these two cohorts are shown in Table 1. According to the established formula, the risk of these patients was evaluated, and the optimal cut-off was equal to 0.313, which is the median CRRS in the training dataset. The detailed information was supplemented in Supplementary Tables 7, 8. The 3- (Figure 3A) and 5-year (Figure 3B) Calibration plots indicated the predicted OS was similar to the ideal survival rates. Kaplan-Meier survival analyses showed the cases with high CRRS exhibited worse survival rates both in the TCGA-BLCA (P < 0.001, Figure 3C) and GSE32894 datasets (P < 0.05, Figure 3D). The receiver operating curves (ROC) of the 1-, 3-, and 5-year OS in the training dataset (Figure 3E) and external validation dataset (Figure 3F) verified the predictive value of the CRRS. Besides, more deaths were observed with the increasing CRRS (Figure 3G, 3H).

**Table 1:** The baseline information of 785 cases enrolled in the present study.

**Parameters**	**TCGA (n=396)**	**GSE32894 (n=224)**
**Survival status**		
Alive	243 (61.3%)	199 (88.8%)
Dead	153 (38.6%)	25 (11.1%)
Follow-up (day)	778.19 ± 814.38	1196.98 ± 767.38
Age	67.84 ± 10.53	69.43 ± 11.28
**Gender**		
Female	104 (26.2%)	61 (27.2%)
Male	292 (73.7%)	163 (72.7%)
**Pathological Stage**		
I	2 (0.5%)	-
II	124 (31.3%)	-
III	138 (34.8%)	-
IV	130 (32.8%)	-
Unknown	2 (0.5%)	-
**pT stage**		
T0	1 (0.2%)	0 (0.0%)
Ta	0 (0.0%)	110 (49.1%)
T1	3 (0.7%)	63 (28.1%)
T2	113 (28.5%)	43 (19.1%)
T3	190 (47.9%)	7 (3.1%)
T4	57 (14.3%)	1 (0.4%)
Unknown	32 (8.0%)	0 (0.0%)
**M stage**		
M0	189 (47.7%)	-
M1	10 (2.5%)	-
Unknown	197 (49.7%)	-
**pN stage**		
N0	229 (57.8%)	27 (12.0%)
N1	44 (11.1%)	3 (1.3%)
N2	75 (18.9%)	10 (4.4%)
N3	7 (1.7%)	0 (0.0%)
Unknown	41 (10.3%)	184 (82.1%)
**Risk stratification**		
High	195 (49.2%)	9 (4.0%)
Low	201 (50.7%)	212 (95.9%)
CRRS	0.41 ± 0.39	0.18 ± 0.081

TCGA, the Cancer Genome Atlas; CRRS, circadian rhythm-related score. The results are shown as mean ± standard deviation (SD).

To screen novel biomarkers, we compared the expression difference of these genes between adjacent normal and BCa tissues utilizing the Wilcoxon signed-rank test (Supplementary Figure 1). In addition, the prognostic values of the 16 genes were also evaluated both in the TCGA-BLCA cohort (Supplementary Table 9 and Supplementary Figure 2) and the GSE32894 cohort (Supplementary Table 10 and Supplementary Figure 3), and X-tile was used to determine the optimal cut-off for Kaplan-Meier analyses [14]. The protein expression level of these genes between normal and BCa samples were also detected via immunohistochemistry (IHC), as supplemented in Supplementary Figure 4.

### The clinical association of CRRS

**Figure 4:**
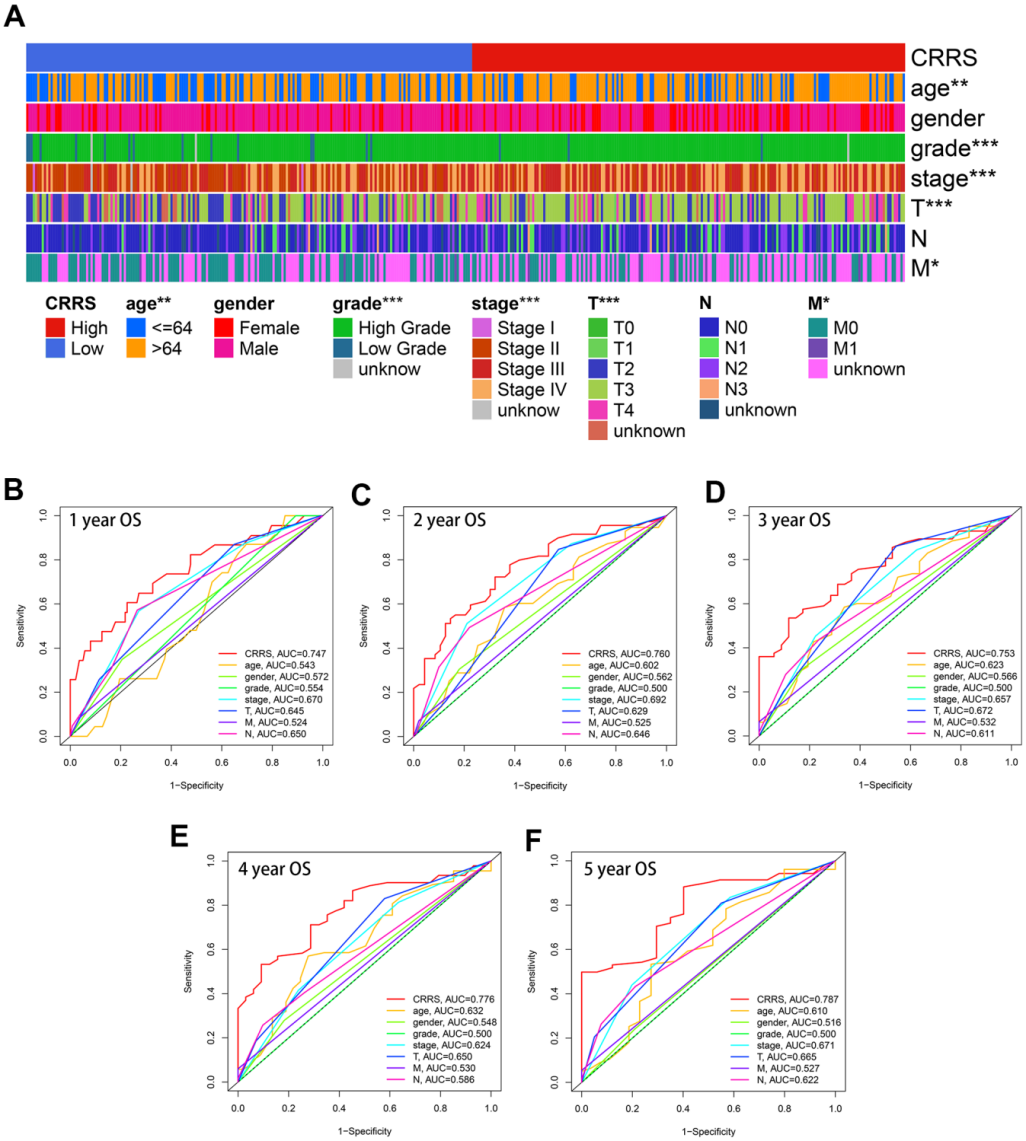
**Clinical association of CRRS.** (**A**) The heatmap indicating CRRS was significantly associated with age, gender, tumor grade, tumor stages, pathological T stages, and M stages utilizing Chi-square tests. (**B**–**F**). The time-dependent ROC analyses indicated the CRRS showed superiority over other clinical features in predicting the 1- (**B**), 2- (**C**), 3- (**D**), 4- (**E**), and 5-year (**F**) overall survival rate. ROC, receiver operating curve; CRRS, circadian rhythm-related score. *, P < 0.05; **, P < 0.01; ***, P < 0.001.

As displayed in Figure 4A, CRRS was significantly correlated with age (P < 0.01), tumor grade (P < 0.001), tumor stage (P < 0.001), pathological T stages (P < 0.001), and M stages (P < 0.05) via Chi-square test after excluding the cases with unknown statuses.

Besides, the CRRS was superior to the clinicopathological features in OS prediction. The univariate (HR = 2.91, P < 0.01) and multivariate (HR = 2.83, P < 0.01) analyses indicated that the CRRS was an independent risk factor after transforming the parameters into binary variables (Table 2). The areas under curve (AUCs) of each variable were calculated and compared. The predictive ability of the CRRS was better than other clinicopathological traits in 1- (AUC = 0.747, Figure 4B), 2- (AUC = 0.760, Figure 4C), 3- (AUC = 0.753, Figure 4D), 4- (AUC = 0.776, Figure 4E), and 5-year (AUC = 0.787, Figure 4F) ROC curves.

**Table 2:** Univariate and multivariate Cox analyses of CRRS.

**Parameters**	**Univariate Cox**		**Multivariate Cox**	
	**HR (95%CI)**	**P value**	**HR (95%CI)**	**P value**
Age (≤64 vs. >64)	1.42 (0.82-2.44)	0.204	1.32 (0.76-2.31)	0.322
Gender (Female vs. Male)	1.58 (0.94-2.66)	0.081	1.54 (0.91-2.61)	0.108
Grade (Low vs. High)	3.64 (0.50-26.53)	0.202	1.17 (0.15-9.30)	0.884
Stage (Stage I-II vs. Stage III-IV)	2.26 (1.15-4.44)	0.017	0.50 (0.15-1.67)	0.259
T (T 1-2 vs. T 3-4)	2.41 (1.26-4.61)	0.008	3.00 (0.99-9.13)	0.053
M (M0 vs. M1)	2.55 (1.02-6.40)	0.045	1.45 (0.54-3.87)	0.457
N (N0 vs. N1-3)	2.33 (1.45-3.78)	0.001	2.07 (1.19-3.63)	0.010
CRRS (Low vs. High)	2.45 (1.49-4.01)	< 0.001	2.32 (1.39-3.85)	0.001

HR, hazard ratio; CI, confidence interval; CRRS, circadian rhythm-related score.

### The tumor immune infiltration profiles of CRRS

**Figure 5:**
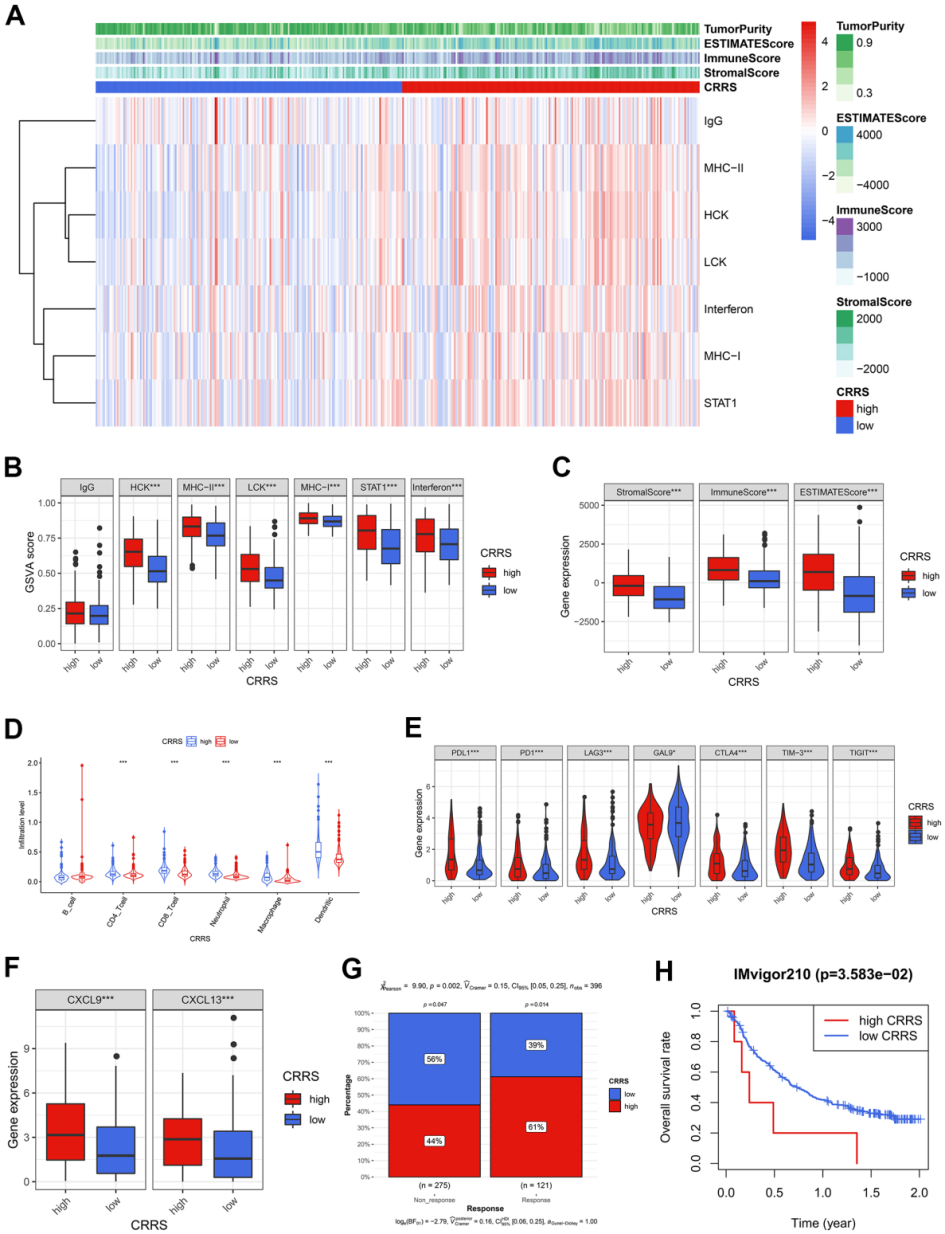
**The tumor immune infiltration and CRRS.** (**A**) The heatmap showed the GSVA scores of 7 immune and inflammatory gene clusters among the BCa patients with high and low CRRS. (**B**) 6 of 7 gene clusters were significantly associated with CRRS via Wilcoxon signed-rank tests. (**C**) The cases with high CRRS carried high Stromal Score, Immune Score, and ESTIMATE Score. (**D**) The patients in the high-CRRS group had a relatively higher infiltration proportion of CD4 T cells, CD8 T cells, neutrophils, macrophages, and dendritic cells. (**E**) The CRRS was positively associated with the expression of routine immune checkpoints, which included PD-L1, PD1, LAG3, GAL9, CTLA-4, TIM-3, and TIGIT. (**F**) The high expression of CXCL9 and CXCL13 was observed in the patients with high CRRS. (**G**) The Chi-square test indicated the high-CRRS patients were more likely to respond to immunotherapy. (**H**) CRRS was also a significant biomarker for prognosis in IMvigor 210 cohort. The optimal cut-off was determined by X-tile software. CRRS, circadian rhythm-related score; GSVA, gene set variation analysis; BCa, bladder cancer.

Seven clusters of immune and inflammatory genes were collected from previous studies, including lgG, HCK, MHC-II, LCK, MHC-I, STAT1, and interferon [15]. Gene Set Variation Analysis (GSVA) was conducted to quantify these immune and inflammatory responses (Supplementary Table 11). The heatmap (Figure 5A) and the boxplots (Figure 5B) showed the CRRS was positively associated with HCK, MHC-II, LCK, MHC-I, STAT1, and interferon. Intratumoral immune heterogeneity might account for the lack of association between CRRS and lgG. Besides, we also evaluated the immune activities with ESTIMATE [16], which was widely used for calculating the proportion of the stromal and immune components in the tumor microenvironment (TME), and the Wilcoxon test displayed the cases with high CRRS carried high tumor infiltration (Figure 5C). Subsequently, the infiltration of different immune cells, including B cells, CD4 T cells, CD8 T cells, neutrophils, macrophages, and dendritic cells, was estimated by the TIMER algorithm [17], and the significant positive association with the CRRS was found except for B cells (Figure 5D).

Given the high immune infiltration and unfavorable prognosis in the high-CRRS patients, we further explored the expression level of immune checkpoints and found that all the collected routine checkpoint genes exhibited significant expression differences (Figure 5E). The high expression level of the immune checkpoints might be responsible for the poor prognosis, implying that the cases with high CRRS were also sensitive to immunotherapy. Hence, some critical biomarkers indicating the immunotherapy efficacy, including CXCL9 [18], CXCL13 [18], and TIDE scores [19], were adopted to evaluate the immunotherapeutic sensitivity. Compared with the cases in the low-CRRS group, the patients in the high-CRRS group had higher expression levels of CXCL9 (P < 0.001) and CXCL13 (P < 0.001, Figure 5F). Th Chi-square test (P < 0.01, Figure 5G) displayed that the patients labelled with high CRRS would be more likely to benefit from the immunotherapy. CRRS could also serve as a prognosis biomarker for IMvigor210 cohort, who have received atezolizumab treatment (P < 0.05, Figure 5H). The calculated CRRSs of IMvigor210 cohort were shown in Supplementary Table 12.

### The association between CRRS and cisplatin response

**Figure 6:**
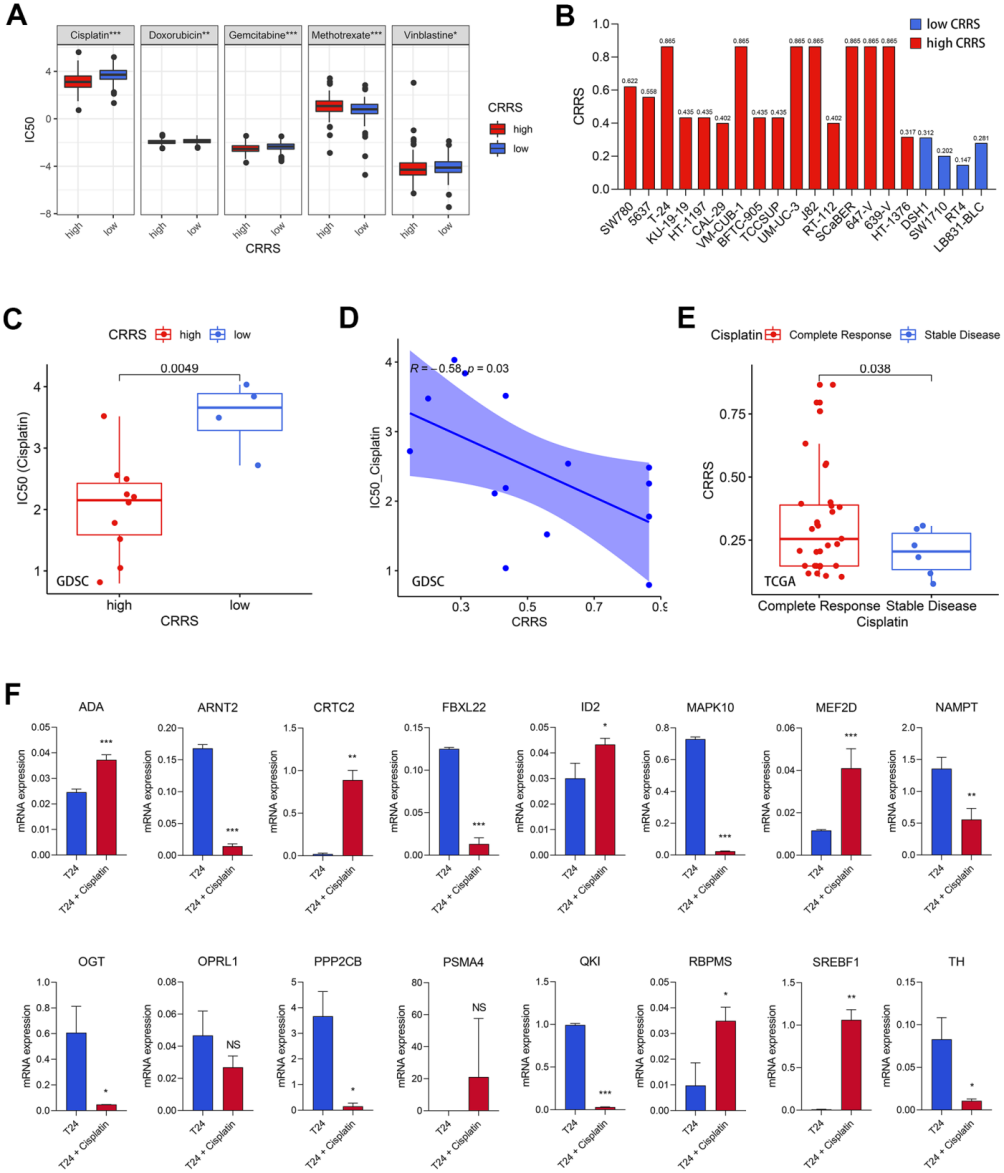
**Cisplatin efficacy and CRRS.** (**A**) The patients with high CRRS were more sensitive to cisplatin, doxorubicin, gemcitabine, and vinblastine, while the CRRS was positively associated with the sensitivity of methotrexate. (**B**) The evaluated CRRS and risk stratification of 20 BCa cell lines. (**C**) The cell lines with low CRRS exhibited high IC50 values via Wilcoxon signed-rank test. (**D**) The Spearman correlation analysis between CRRS and IC50 values among 20 BCa cell lines. (**E**) The patients with complete response had higher CRRS than those with stable disease. (**F**) The mRNA expression of the 16 CRRS genes after the treatment with 20μM cisplatin in T24 cells. CRRS, circadian rhythm-related score; IC50, half inhibitory concentration; BCa, bladder cancer.

Based on the predicted half inhibitory concentration (IC50) of the patients from TCGA, we found the CRRS was significantly associated with the sensitivity of common chemotherapeutic agents, such as cisplatin (P < 0.001), doxorubicin (P < 0.01), gemcitabine (P < 0.001), methotrexate (P < 0.001), and vinblastine (P < 0.05, Figure 6A). Meanwhile, we downloaded the transcriptome expression values of 20 BCa cell lines and corresponding IC50 of the common chemotherapeutic drugs from Genomics of Drug Sensitivity in Cancer (GDSC, https://www.cancerrxgene.org/) database [20]. The CRRS of each cell line was evaluated, and the risk stratification was based on the median CRRS in the TCGA-BLCA cohort, which was mentioned above (Supplementary Table 13 and Figure 6B). We showed the cells with high CRRS exhibited a low IC50 of cisplatin (P < 0.01, Figure 6C), while no significance was achieved in doxorubicin (P > 0.05), gemcitabine (P > 0.05), methotrexate (P > 0.05), and vinblastine (P > 0.05, Supplementary Figure 5). The Spearman correlation analysis indicated the CRRS was tightly associated with the IC50 values (r = -0.58, P < 0.05, Figure 6D). The collected cisplatin response of TCGA-BLCA patients was also retrieved to serve as the clinical sample validation, and the cases reported to carry complete response to cisplatin have a significantly higher CRRS than the patients with stable disease (P < 0.05, Figure 6E).

Afterwards, the experimental validation was also conducted to verify the conclusion. We treated T24 cells with 20 μM cisplatin for 24 hours, which was reported in previous studies [21], to detect the expression difference of the 16 genes in the risk model. The real-time quantitative PCR (RT-qPCR) results were shown in Figure 6F, and the primers were supplemented in Supplementary Table 14. It was found that ADA (P < 0.001), CRTC2 (P < 0.001), ID2 (P < 0.05), MEF2D (P < 0.001), RBPMS (P < 0.05), and SREBF1 (P < 0.01) were significantly up-regulated in the T24 BCa cells treated with cisplatin, while ARNT2 (P < 0.001), FBXL22 (P < 0.001), MAPK10 (P < 0.001), NAMPT (P < 0.01), OGT (P < 0.05), PPP2CB (P < 0.05), QKI (P < 0.001), and TH (P < 0.05) were obviously decreased. Most of the 16 genes have significant expression differences after the treatment with cisplatin, re-validating the CRRS implicated cisplatin response.

### Gene set variation analysis and gene set enrichment analysis

**Figure 7:**
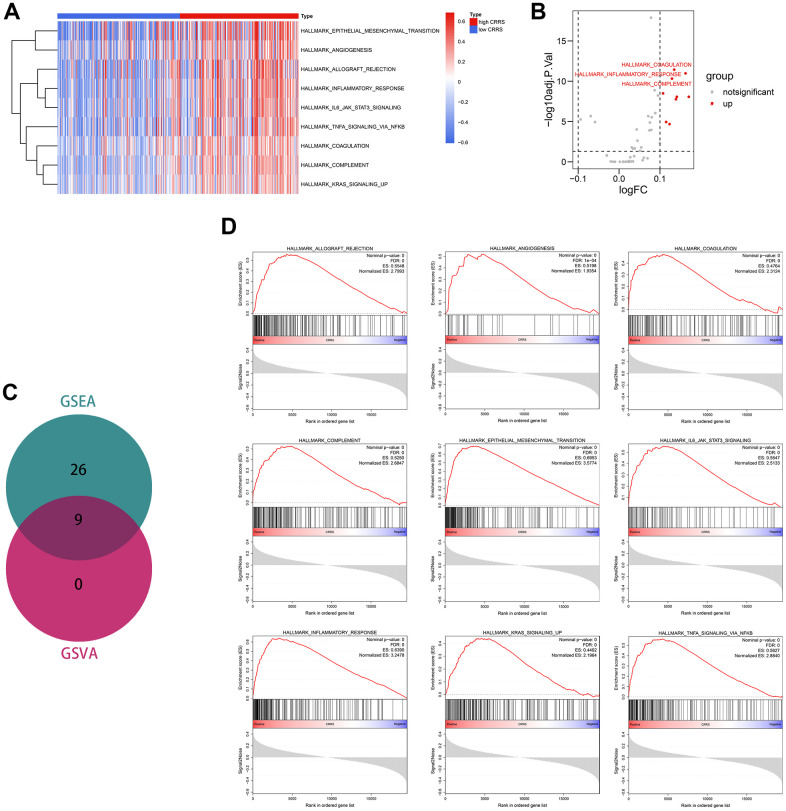
**Functional enrichment analyses.** (**A**) The heatmap displaying the significant phenotypes associated with CRRS. (**B**) A sum of 9 hallmarks was identified with the limma package. (**C**) Venn plot showing 9 phenotypes were overlapped from GSEA and GSVA analyses. (**D**) The overlapped 9 phenotypes included allograft rejection, angiogenesis, coagulation, complement, epithelial-mesenchymal transition, IL6-JAK-STAT3 signaling, inflammatory response, KRAS signaling, and TNFα signaling via NFKB. CRRS, circadian rhythm-related score; GSEA, gene set enrichment analysis; GSVA, gene set variation analysis.

To detect the vital tumor phenotypes correlated with the CRRS, gene set enrichment analysis (GSEA) and GSVA were both performed. Through GSVA analysis, a sum of 9 hallmarks was identified, as shown in Supplementary Table 15 and Figure 7A, 7B. GSEA analysis screened 26 important tumor phenotypes, where the 9 hallmarks were also included (Supplementary Tables 16, 17 and Figure 7C). The details of the 9 overlapped hallmarks are illustrated in Figure 7D.

## DISCUSSION

At the molecular level, the circadian rhythm is formed by the oscillation of the clock gene to produce an autonomous rhythm. Circadian rhythm could regulate many biological processes, such as cell proliferation, cellular metabolism, and hormone secretion, which were the underlying mechanisms of circadian rhythm disorder in tumor initiation and progression [22]. Circadian rhythm dysregulation was often accompanied by the alternation of clock gene expression, which disrupted the normal cell cycle and thus directly promoted tumor cell proliferation [23, 24]. Melatonin, acting as a critical hormone regulating circadian rhythm, was significantly associated with the risk of breast cancer, lung cancer, and cervical carcinoma from previous evidence-based medical researches [25]. The findings above suggested circadian rhythm played an important role in tumorigenesis and tumor development. However, no circadian rhythm-related signature has been constructed in BCa, which is beneficial for personalized management and screening of new biomarkers.

The present study collected the circadian rhythm-related genes from MSigDB, and accordingly, 396 BCa patients were grouped into two clusters. We found the clustering was significantly associated with overall survival rate (P < 0.01) and many other risk clinical parameters, enlightening us to develop a circadian rhythm-related signature to identify significant biomarkers. To make the risk model widely appliable for the samples tested by RNA-seq, microarray, or RT-qPCR, a gene-pair strategy was adopted to construct the prognostic model based on the circadian rhythm-related genes which were differentially expressed between adjacent normal and BCa tissues. After Lasso and univariate Cox regression, a sum of 10 gene pairs was identified, 8 of which were included in the risk model via multivariate Cox regression with stepwise. According to the risk model, the risk of all BCa patients enrolled, including 396 cases from TCGA and 224 cases from GSE32894, was quantified as circadian rhythm-related score, or CRRS. CRRS was a promising predictive tool for BCa prognosis, which was validated in different independent cohorts. Besides, CRRS was superior to other clinicopathological traits in OS evaluation.

The regulation of circadian rhythm to the immune system has been described [26–28]. The inflammation indicators in serum, such as TNF-α, IL-10, and C-reactive protein (CRP), were significantly increased in the subjects with circadian rhythm disorder [29]. Meanwhile, inflammatory factors could also influence the expression of core clock genes. Abreu et al. have found that the expression of BMAL1, PER2, and REV-ERB-α was obviously up-regulated in the Hodgkin lymphoma cells treated with TNF-α [30]. Previous researches suggested that circadian rhythm and immune system could influence each other. However, how circadian rhythm influenced the TME remains unclear. Here, we found the patients with high CRRS carried high immune infiltration and high checkpoint gene expression, which might account for the poor prognosis, and thus be more likely to benefit from immunotherapy. We screened some important biomarkers, which might be the cut-in points in future studies.

Cisplatin-based neoadjuvant chemotherapy remains one of the dominant medical treatments in BCa for the moment. Regarding how circadian rhythm affects cisplatin efficacy, several studies have been published. For instance, Wang et al. have found PER2, a circadian clock gene, enhanced the effect of cisplatin by suppressing PI3K/Akt pathway in ovarian cancer cells [31]. It was reported that circadian gene TIMELESS could decrease the cisplatin sensitivity by activating the Wnt/β-catenin pathway [32]. Besides, some researchers held that circadian rhythm was closely associated with DNA repair function and thus could influence cisplatin sensitivity since cisplatin serves as a DNA damaging agent [33]. Given the findings above, we explored the association between CRRS and cisplatin efficacy based on TCGA and GDSC databases and found CRRS was a promising clinical tool to evaluate the cisplatin response. The 16 genes, which comprised CRRS, mostly showed expression differences in T24 cells with cisplatin treatment. The results above re-validated the tight association between circadian rhythm and cisplatin and provided some important biomarkers.

Some genes comprising CRRS have been reported to involve the malignant phenotypes in different cancers, such as CRTC2 [34], FBXL22 [35], OPRL1 [36], and PSMA4 [37]. Though the genes mentioned above mainly were differentially expressed between BCa and adjacent normal tissues (Supplementary Figure 2) and showed significant predictive values for prognosis (Supplementary Figures 3, 4), their functions in BCa have not been reported. Totally, the proposed model was helpful to identify novel biomarkers, providing cut-in points for further experimental researches.

However, the limitations of the present study should not be neglected. First, the research is retrospective, and a large-scale, multi-center, and prospective study was demanded to validate the clinical usefulness of CRRS. Second, some important phenotypes were associated with the CRRS via bioinformatical analyses and big-data mining, and the experimental validation would be helpful.

In conclusion, a novel circadian rhythm-related signature was proposed, providing a useful tool to evaluate tumor immune infiltration, cisplatin efficacy, and prognosis in BCa.

## MATERIALS AND METHODS

### Data collection and processing

The transcriptome RNA sequencing data in count and FPKM format and corresponding clinical information were obtained from TCGA (https://portal.gdc.cancer.gov/) as the training dataset. GSE32894 dataset, which included the transcriptome data and clinicopathological features of 224 BCa cases, was directly downloaded from GEO (https://www.ncbi.nlm.nih.gov/geo/) as the external validation cohort. The Ensemble IDs and probe IDs were transformed into gene symbols according to the corresponding annotation files downloaded from the GENECODE (version 22, GRCh38) and GEO. The genes with average expression < 0.5 were excluded from the present study. The gene expression data with FPKM format and the prognosis information of 348 patients with metastatic urothelial carcinoma of IMvigor210 cohort were obtained from IMvigor210CoreBiologies package in R (version 3.6.3). EdgeR package of R was used for genomic difference detection with |logFC| > 1 and adjusted P < 0.05 filtering. The volcano plot was drawn with the ggplot2 package to visualize the difference analysis.

### Unsupervised clustering

The consensus clustering was conducted to identify the circadian subtypes of BCa through the ConsensusClusterPlus R package. We identified the optimal k value, equal to the clustering number, with the nmf R package. The slowest rising line in the cumulative distribution function (CDF) curve represented the best k value.

### Survival analysis

To avoid including the deaths caused by surgical injury in the study, the cases with the following duration < 30 days were ruled out. The Kaplan-Meier survival analysis with log-rank test was performed with survival package of R. Survival R package was also used for univariate and multivariate Cox regression. Lasso was conducted by the glmnet R package, and 10-fold cross-validation was performed. The calibration plots were drawn with the rms package. The time-dependent ROC curves were completed with the survivalROC package.

### Gene-pair strategy

Here, we utilized a gene-pair strategy to make the predictive model achieve broad applicability. We defined a novel combination of gene A and gene B, or A|B, as 1 when the expression value of A was higher than that of B; otherwise, it would be regarded as 0. The screened genes were cyclically paired, and a 0-or-1 matrix was successfully established after excluding the gene pairs with < 20% proportion of 0 or 1 in the training dataset.

### Evaluation of immune infiltration

The ESTIMATE algorithm was used to evaluate the immune and stromal components ratios in TME, quantified as the Immune Score and Stromal Score [16]. The ESTIMATE Score represented the sum of the Immune Score and Stromal Score. The infiltration proportion of immune cells, including B cells, CD4 T cells, CD8 T cells, neutrophils, macrophages, and dendritic cells, were estimated on the TIMER website (http://cistrome.dfci.harvard.edu/TIMER/). The immunotherapeutic response was predicted with the TIDE algorithm, which offered an official website (http://tide.dfci.harvard.edu/).

### The chemotherapeutic effectiveness analyses

The chemotherapeutic sensitivity of BCa patients in the TCGA-BLCA cohort was evaluated through the pRRophetic R package [38]. The transcriptome data and the IC50 values of 20 different BCa cell lines were retrieved from the GDSC database (https://www.cancerrxgene.org/) to confirm the predictive value of CRRS to chemotherapy response. The microarray RNA expression data from the GDSC dataset was normalized with Robust Multi-Array Average (RMA). The information about the response statuses to cisplatin among the BCa cases was also downloaded from TCGA, including complete response (CR), partial response (PR), clinical progressive disease (PD), and stable disease (SD).

### GSEA and GSVA

The hallmark gene sets v. 7.2 was downloaded from MSigDB as the reference dataset. GSVA was conducted with the GSVA package of R, and the parameters were set as follows: min. size = 10, max. size =500, verbose = Ture, and parallel. size = 1. Limma was used for the difference detection, and the filtering threshold was set as |logFC| > 0.1 and adjusted P < 0.05. GSEA was performed with GSEA software (version 4.1.0), and the number of permutations was set to 1000. The gene sets with nominal P < 0.05 and FDR q <0.05 were considered to be statistically significant.

### Cell culture and treatment

The T24 cell line was purchased from Shanghai Institutes for Biological Sciences (Shanghai, China) and maintained in the McCoy’s 5 A Medium (Gibco, USA) supplemented with 1% antibiotics and 10% fetal bovine serum (Gibco, USA). The cells were cultured in a humidified atmosphere with 5% CO2 at 37° C. The cells were treated with 20μM cisplatin (Sigma-Aldrich, USA) for 24 hours.

### RT-qPCR

The total RNA of the T24 cells were collected employing Trizol (ThermoFisher Scientific, Germany). Subsequently, PrimeScript RT Reagent Kit (Takara, China) and SYBR Premix ExTaq kit (Takara, China) were used to synthesize and amplify the cDNA. The ABI Prism 7000 system (Applied Biosystems, USA) helped identify the mRNA expression level, and the data were normalized with the 2-ΔΔC method.

### Immunohistochemistry

The immunohistochemical staining of the CRRS genes was collected from The Human Protein Atlas (version 20.1; https://www.proteinatlas.org/), a comprehensive database for detecting the protein distribution and expression in human normal and tumor tissues.

### The statistical analysis

We utilized R software (version 3.6.3) to conduct the statistical analysis. The student’s t-test was utilized to compare the difference of the continuous variables obtained from vitro experiments. At the same time, the Wilcoxon Signed-rank test was adopted for the continuous variables collected from bioinformatical analyses. The violin diagrams and the boxplots were also drawn with the ggplot2 package. The Chi-square test was used for categorical variables, and the results were visualized with the ggplot2 and the ggstatsplot packages.

## Supplementary Material

Supplementary Figures

Supplementary Table 1

Supplementary Table 2

Supplementary Table 3

Supplementary Table 4

Supplementary Tables 5 and 6

Supplementary Table 7

Supplementary Table 8

Supplementary Tables 9 and 10

Supplementary Table 11

Supplementary Table 12

Supplementary Tables 13 and 14

Supplementary Table 15

Supplementary Table 16

Supplementary Table 17
