# Supplementary material for: A circadian rhythm-related gene signature associated with tumor immunity, cisplatin efficacy, and prognosis in bladder cancer
Source: Aging (Albany NY). 2021 Dec 3;13(23):25153–79. doi: 10.18632/aging.203733 (PMC8714136; doi:10.18632/aging.203733)
Supplement: Supplementary Figures [file aging-13-203733-s001.pdf]

SUPPLEMENTARY FIGURES

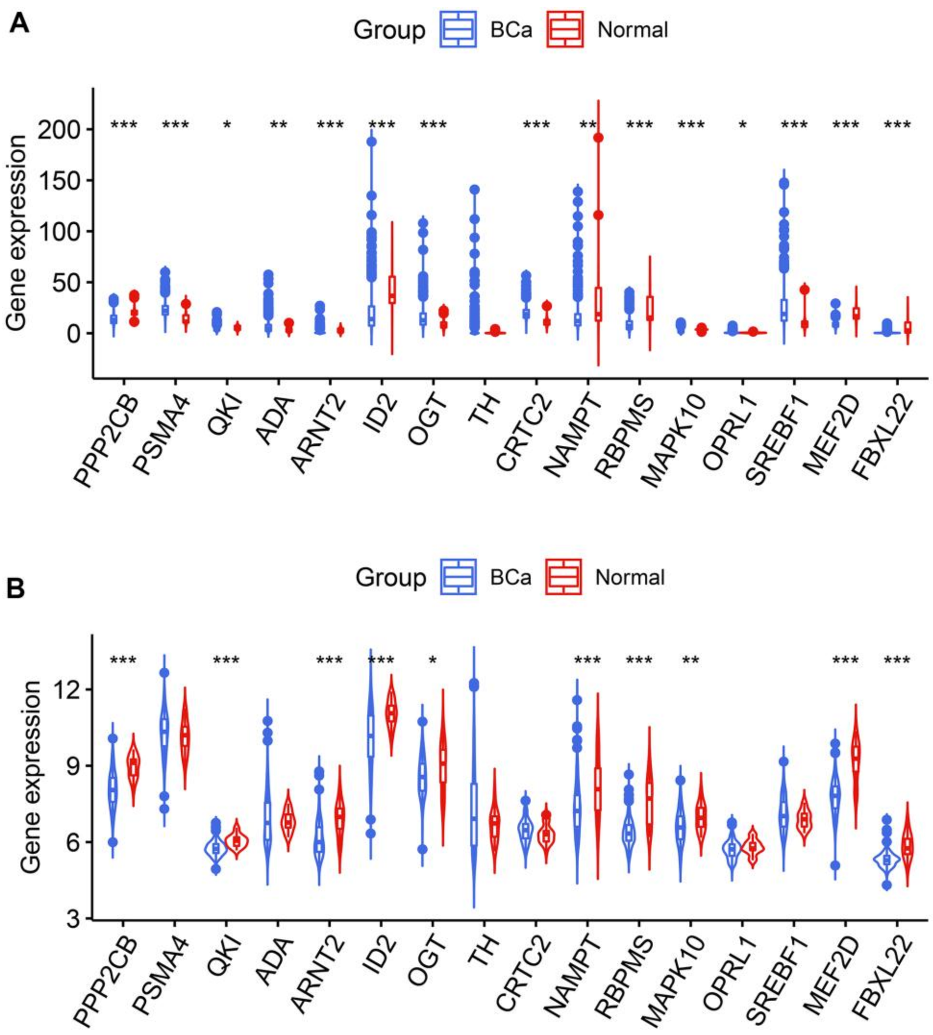

Supplementary Figure 1. The expression differences of the 16 CRRS genes between adjacent normal and BCa samples in the TCGA cohort (**A**) and GSE32894 cohort (**B**) via Wilcoxon signed-rank tests.

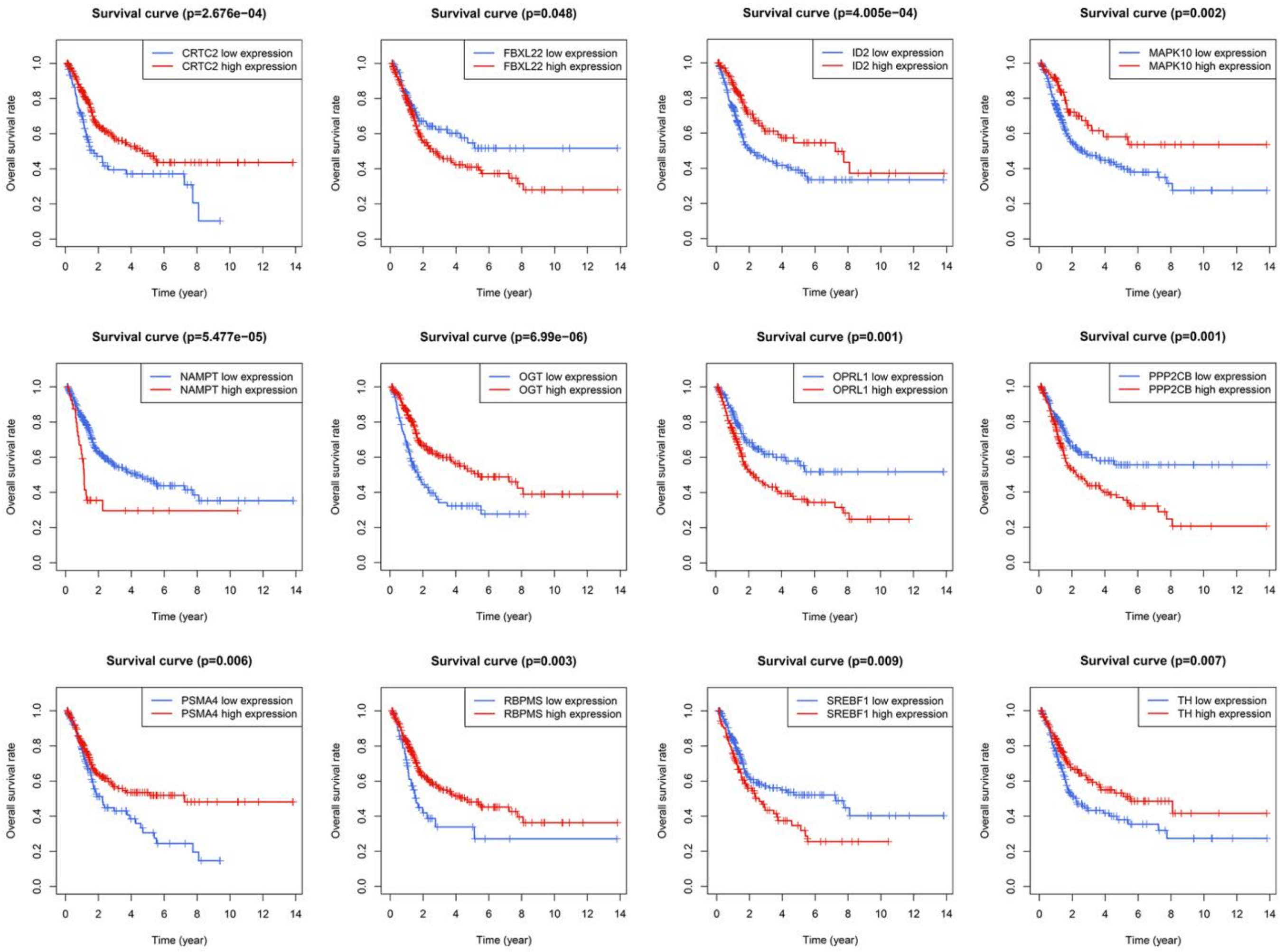

Supplementary Figure 2. The prognostic values of the 16 CRRS genes in the TCGA cohort.

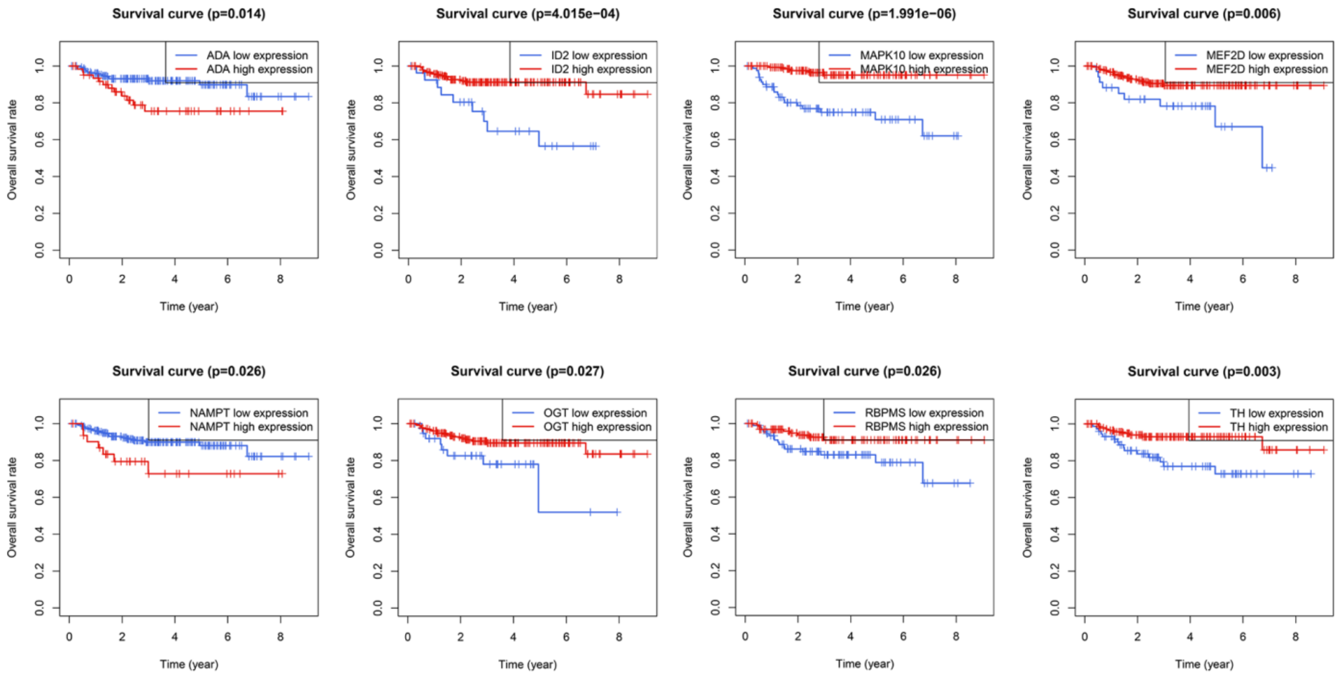

Supplementary Figure 3. The prognostic values of the 16 CRRS genes in the GSE32894 cohort.

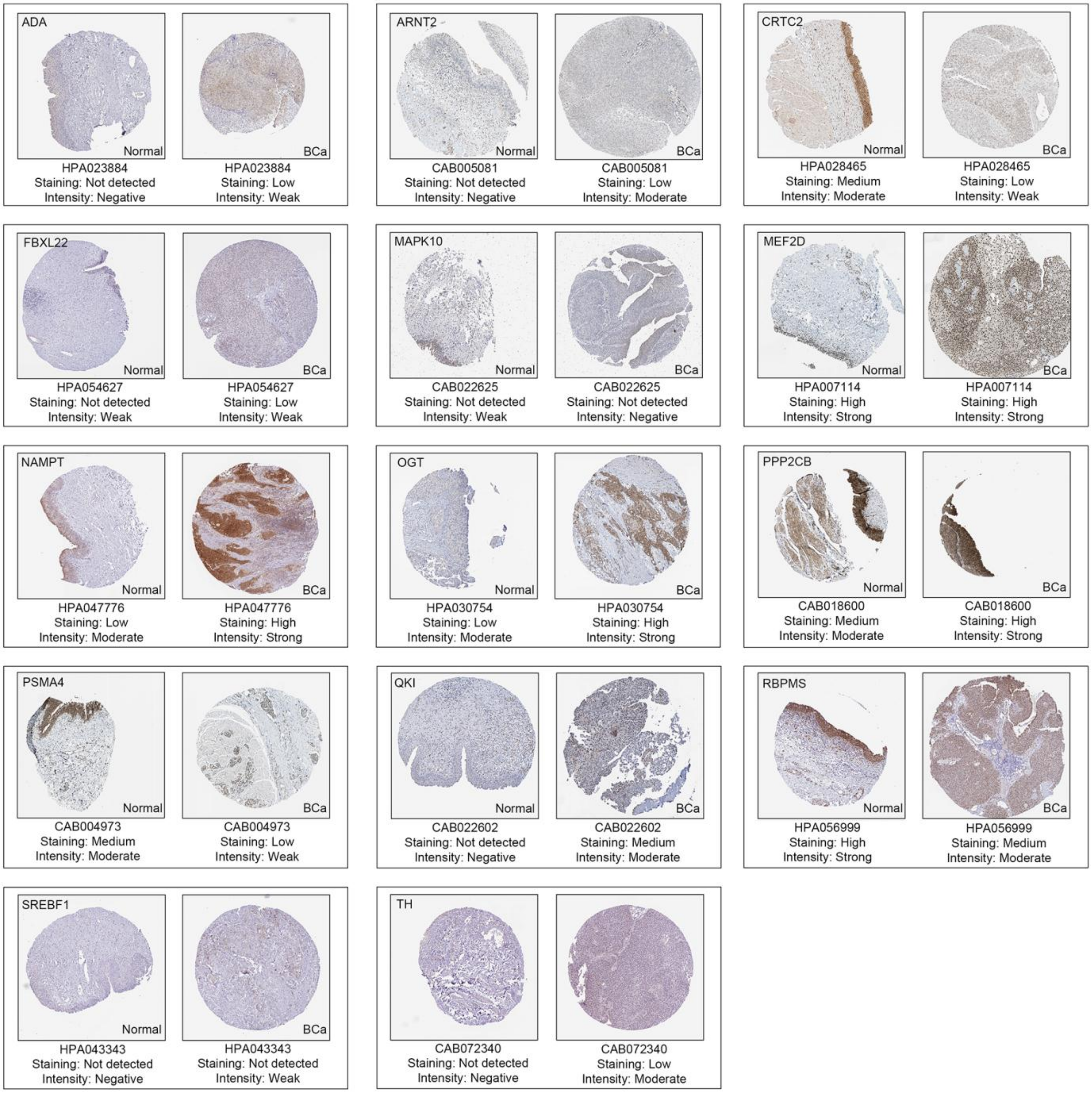

Supplementary Figure 4. The immunohistochemical staining of the CRRS genes in human normal and BCa tissues.

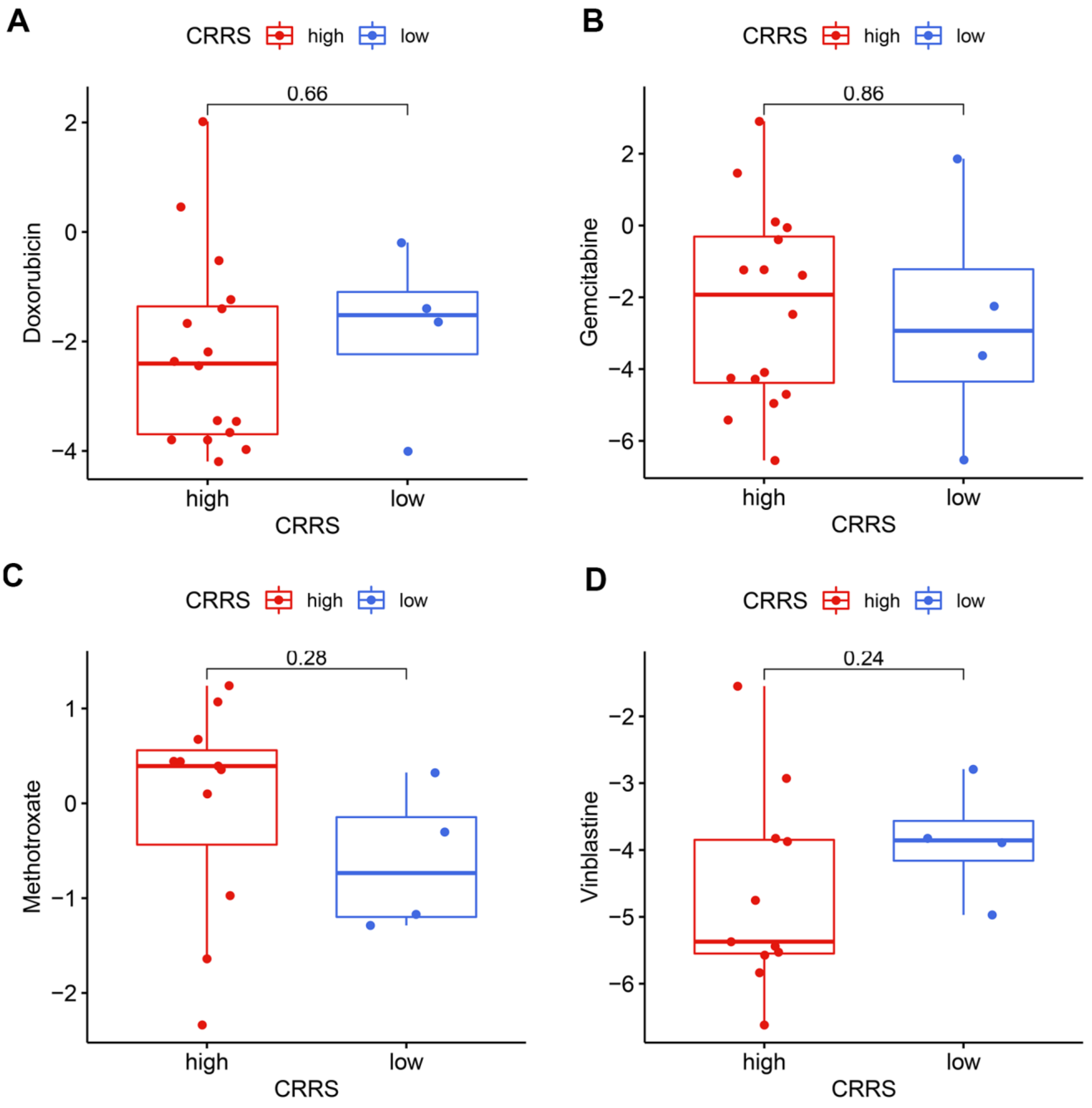

Supplementary Figure 5. The association between CRRS and the IC50 values of doxorubicin (**A**), gemcitabine (**B**), methotrexate (**C**), and vinblastine (**D**) in the BCa cell lines.
