# Supplementary material for: A circadian rhythm-related gene signature associated with tumor immunity, cisplatin efficacy, and prognosis in bladder cancer
Source: Aging (Albany NY). 2021 Dec 3;13(23):25153–79. doi: 10.18632/aging.203733 (PMC8714136; doi:10.18632/aging.203733)
Supplement: Supplementary Tables 5 and 6 [file aging-13-203733-s006.pdf]

Supplementary Table 5. The details of the risk model by means of multivariate Cox regression with stepwise.

id	coef	HR	HR.95L	HR.95H	pvalue
PPP2CB|CRTC2	0.313859881	1.368697943	0.954190951	1.96326957	0.088156685
PSMA4|NAMPT	-0.546556531	0.578939944	0.393937839	0.850823215	0.005396217
QKI|RBPMS	0.329671729	1.390511589	0.972509447	1.988178609	0.070737473
ADA|MAPK10	0.316172646	1.371867083	0.882657423	2.132219412	0.15995725
ARNT2|OPRL1	-0.687965343	0.502597643	0.363026562	0.695828948	0.000034
ID2|SREBF1	-0.437329062	0.645758902	0.455211219	0.916068282	0.014232299
OGT|MEF2D	-0.558600847	0.572008832	0.40672795	0.804454437	0.001324701
TH|FBXL22	-0.350313151	0.704467451	0.493085274	1.006467675	0.054286136

Supplementary Table 6. The spearman correlation coefficients between the 16 CRRS genes and circadian transcription factors.

TF	CRRS_gene	Spearman_correlation	pvalue
PER1	PPP2CB	0.282	5.78093075573597E-09
PER1	PSMA4	-0.065	0.189990010775076
PER1	QKI	0.219	0.0000073556873937004
PER1	ADA	0.209	0.0000186748072864482
PER1	ARNT2	-0.08	0.107097529553661
PER1	ID2	-0.018	0.721606561422284
PER1	OGT	-0.156	0.00151541828986829
PER1	TH	-0.262	6.9347012076925E-08
PER1	CRTC2	-0.005	0.922923590920486
PER1	NAMPT	0.277	1.19788307528409E-08
PER1	RBPMS	0.014	0.77648121597283
PER1	MAPK10	-0.162	0.0010125040968491
PER1	OPRL1	0.096	0.052485601947928
PER1	SREBF1	-0.07	0.156794917100766
PER1	MEF2D	0.48	4.98238207011712E-25
PER1	FBXL22	0.097	0.048404465245423
ARNTL	PPP2CB	0.286	3.64444944036374E-09
ARNTL	PSMA4	0.054	0.274365487857749
ARNTL	QKI	0.312	9.23587578673394E-11
ARNTL	ADA	0.304	3.32072102294723E-10
ARNTL	ARNT2	0.014	0.7774721796168
ARNTL	ID2	-0.176	0.000345442760082285
ARNTL	OGT	0.042	0.400897836235977
ARNTL	TH	-0.262	6.79031747327374E-08
ARNTL	CRTC2	-0.06	0.221922108953692
ARNTL	NAMPT	0.345	5.98192804606054E-13
ARNTL	RBPMS	-0.187	0.000142868750526303
ARNTL	MAPK10	-0.114	0.0212632782405979
ARNTL	OPRL1	0.183	0.000193893613183833
ARNTL	SREBF1	-0.047	0.337795375026129
ARNTL	MEF2D	0.297	8.00068086478635E-10
ARNTL	FBXL22	-0.085	0.0834569272544813
CLOCK	PPP2CB	0.323	1.98215465907806E-11
CLOCK	PSMA4	-0.005	0.914261633994987
CLOCK	QKI	0.317	4.82035133174775E-11
CLOCK	ADA	-0.045	0.366248093309971
CLOCK	ARNT2	0.166	0.000732603609458834
CLOCK	ID2	-0.033	0.49877762642488
CLOCK	OGT	0.113	0.0221696188543565
CLOCK	TH	-0.047	0.341038087379232
CLOCK	CRTC2	-0.074	0.135200126646786
CLOCK	NAMPT	0.396	6.93387354272534E-17
CLOCK	RBPMS	-0.101	0.0415707788595762
CLOCK	MAPK10	0.184	0.000171536398851449
CLOCK	OPRL1	-0.042	0.394053495180797
CLOCK	SREBF1	0.096	0.0519505258236541
CLOCK	MEF2D	0.203	0.0000352825690784988

CLOCK	FBXL22	-0.102	0.0393201635250358
PER2	PPP2CB	0.364	2.76579194910235E-14
PER2	PSMA4	-0.13	0.00819041404470939
PER2	QKI	0.196	0.0000624473549864017
PER2	ADA	-0.004	0.928426331367054
PER2	ARNT2	0.064	0.195850407221862
PER2	ID2	0.024	0.622194590931014
PER2	OGT	0.065	0.190822565288749
PER2	TH	-0.21	0.0000172810300478247
PER2	CRTC2	-0.194	0.0000736024601316075
PER2	NAMPT	0.386	4.68094755849204E-16
PER2	RBPMS	0.076	0.126229351152509
PER2	MAPK10	0.156	0.00147065559316406
PER2	OPRL1	-0.058	0.237002835558395
PER2	SREBF1	0.026	0.603929368408065
PER2	MEF2D	0.29	2.22340540332294E-09
PER2	FBXL22	-0.013	0.787736262994609
