# Supplementary material for: A circadian rhythm-related gene signature associated with tumor immunity, cisplatin efficacy, and prognosis in bladder cancer
Source: Aging (Albany NY). 2021 Dec 3;13(23):25153–79. doi: 10.18632/aging.203733 (PMC8714136; doi:10.18632/aging.203733)
Supplement: Supplementary Tables 9 and 10 [file aging-13-203733-s009.pdf]

Supplementary Table 9. The P values of the 16 CRRS genes via Kaplan-Meier survival analysis in the TCGA cohort.

Gene	pvalue
PPP2CB	0.00119351373210486
PSMA4	0.00553335180691217
QKI	0.0531965137366481
ADA	0.145667391494244
ARNT2	0.0875637046442688
ID2	0.000400456719568121
OGT	6.99019535410628E-06
TH	0.00739078078934785
CRTC2	0.000267642081746122
NAMPT	0.0000547669235902992
RBPMS	0.00278692797305302
MAPK10	0.00170324171058989
OPRL1	0.00100260158060006
SREBF1	0.00886033858742974
MEF2D	0.0836396104281839
FBXL22	0.0484398887539246

Supplementary Table 10. The P values of the 16 CRRS genes via Kaplan-Meier survival analysis in the GSE32894 cohort.

Gene	pvalue
PPP2CB	0.19840575657814
PSMA4	0.0512927853392764
QKI	0.0737557610829734
ADA	0.0137360739475768
ARNT2	0.212478890791293
ID2	0.000401526258070994
OGT	0.0268431273486096
TH	0.00336694319562603
CRTC2	0.448685428580084
NAMPT	0.026193748611878
RBPMS	0.0263911073223753
MAPK10	1.99072940310074E-06
OPRL1	0.060364305328372
SREBF1	0.128023643497365
MEF2D	0.00565422501840018
FBXL22	0.625931543379857
