# Supplementary material for: A circadian rhythm-related gene signature associated with tumor immunity, cisplatin efficacy, and prognosis in bladder cancer
Source: Aging (Albany NY). 2021 Dec 3;13(23):25153–79. doi: 10.18632/aging.203733 (PMC8714136; doi:10.18632/aging.203733)
Supplement: Supplementary Tables 13 and 14 [file aging-13-203733-s012.pdf]

Supplementary Table 13. The IC50 values and calculated CRRS of the 20 BCa cell lines.

id	CRRS	Risk	Cisplatin	Methotrexate	Doxorubicin	Vinblastine	Gemcitabine
SW780	0.6218074	high	2.5401121	-2.33514264	2.0186005	-5.834158	2.90442625
5637	0.5583428	high	1.5233729	-1.63878302	-3.7970998	-6.615991	-1.2320148
T_24	0.8646304	high	NA	NA	-1.2347614	NA	-0.05737397
KU_19_19	0.4345612	high	2.1894818	-0.971251	0.4585119	-3.870262	1.46146064
HT_1197	0.4345612	high	3.5162574	0.44139082	-1.4008072	-1.548121	-0.39201549
CAL_29	0.4015377	high	2.1125156	0.3566166	-2.1881597	-5.527362	-4.09075495
VM_CUB_1	0.8646304	high	NA	NA	-2.4430689	NA	-4.27746705
BFTC_905	0.4345612	high	1.0370687	0.09939585	-4.19149	-5.570882	-6.54221767
TCCSUP	0.4345612	high	NA	NA	-0.5197181	NA	0.10590606
UM_UC_3	0.8646304	high	1.7794393	0.67527913	-2.3634623	-4.751309	-4.24944117
J82	0.8646304	high	NA	NA	-3.6614741	NA	-4.70299634
RT_112	0.4015377	high	NA	NA	-3.795467	NA	-5.41436675
SCaBER	0.8646304	high	2.484372	1.07195731	-3.4567252	-5.439199	-2.47287178
647_V	0.8646304	high	2.2538245	0.3929999	-3.4446805	-3.825809	-4.9542453
639_V	0.8646304	high	0.7969217	0.44358272	-3.9721519	-5.371709	-1.38215512
HT_1376	0.3167663	high	NA	1.23989051	-1.6657156	-2.927075	-1.2352954
DSH1	0.3124189	low	3.8393146	-1.28696771	-1.6432175	-3.823059	-2.24507309
SW1710	0.2018119	low	3.4772703	0.32408962	-4.004044	-4.970615	-6.5276792
RT4	0.1471075	low	2.7182992	-1.16844661	-0.1939384	-2.791072	1.86036582
LB831_BLC	0.2806218	low	4.0320381	-0.30205164	-1.3963335	-3.890481	-3.62147262

Supplementary Table 14. The sequence of the primers used in the present study.

Gene symbol	ForwardPrimer(5'->3')	ReversePrimer(5'->3')
GAPDH	GGAGCGAGATCCCTCCAAAAT	GGCTGTTGTCATACTTCTCATGG
PPP2CB	CTGAACGAGAACCAAGTGCG	ACGAACCTCTTGCACATTTGA
PSMA4	AGTGTGGCAGGCATAACTTCT	TCACAAGGTATTGGCTCCTGA
QKI	AAGCCCACCCCAGATTACCT	ACTCTGCTAATTTCTTCGTCCAG
ADA	GCCTTCGACAAGCCCAAAGTA	CTCTGCTGTGTTAGCTGGGAG
ARNT2	ATGGCGCGTACAAGCCTTC	AGTCAGACACATAAATCACTCGC
ID2	AGTCCCGTGAGGTCCGTTAG	AGTCGTTCATGTTGTATAGCAGG
OGT	TCCTGATTTGTACTGTGTTCGC	AAGCTACTGCAAAGTTCGGTT
TH	GGAAGGCCGTGCTAAACCT	GGATTTTGGCTTCAAACGTCTC
CRTC2	CCGGTTACAGGCCCAAAAACT	AATGTGGCGGGTGTATCGG
NAMPT	CGGCAGAAGCCGAGTTCAA	GCTTGTGTTGGGTGGATATTGTT
RBPMS	AAACAGCCTGTAGGTTTTGTCA	GGAATTTCAGGATCGAAGCGG
MAPK10	CAGATGGAATTAGACCATGAGCG	TCAATGTGCAATCAGACTTGACT
OPRL1	TTCTGGGAGGTTATCTACGGC	GGATGACGTACATGACAAGGC
SREBF1	ACAGTGACTTCCCTGGCCTAT	GCATGGACGGGTACATCTTCAA
MEF2D	CCAGCGAATCACCGACGAG	GCAGTCACATAGCACGCTC
FBXL22	CCATGCACATAACCCAGCTCA	CCGAGGTGATTTCGGTCCAAC
