# Supplementary material for: A circadian rhythm-related gene signature associated with tumor immunity, cisplatin efficacy, and prognosis in bladder cancer
Source: Aging (Albany NY). 2021 Dec 3;13(23):25153–79. doi: 10.18632/aging.203733 (PMC8714136; doi:10.18632/aging.203733)
Supplement: Supplementary Table 17 [file aging-13-203733-s015.pdf]

Supplementary Table 17. The GSEA results in the subgroup with low CRRS.

NAME	GS<br>follow link to MSigDB	GS DETAILS	SIZE	ES	NES	NOM p-val	FDR q-val	FWER p-val	RANK AT MAX	LEADING EDGE
HALLMARK_ OXIDATIVE_ PHOSPHORYLATION	HALLMARK_ OXIDATIVE_ PHOSPHORYLATION	Details ...	185	-0.32739022	-1.4821173	0.004026846	0.0662548	0.288	3605	tags=27%, list=18%, signal=33%
HALLMARK_ BILE_ACID_ METABOLISM	HALLMARK_ BILE_ACID_ METABOLISM	Details ...	112	-0.29618245	-1.2587848	0.09154929	0.29479158	0.937	4446	tags=33%, list=23%, signal=43%
HALLMARK_ PEROXISOME	HALLMARK_ PEROXISOME	Details ...	104	-0.29905438	-1.2510827	0.10557185	0.20794153	0.95	4667	tags=33%, list=24%, signal=43%
HALLMARK_ DNA_REPAIR	HALLMARK_ DNA_REPAIR	Details ...	148	-0.26814818	-1.1834313	0.15096954	0.2667444	0.994	5692	tags=38%, list=29%, signal=53%
HALLMARK_ FATTY_ACID_ METABOLISM	HALLMARK_ FATTY_ACID_ METABOLISM	Details ...	156	-0.26005426	-1.1436396	0.2021858	0.28168854	0.998	2758	tags=21%, list=14%, signal=24%
HALLMARK_ ADIPOGENESIS	HALLMARK_ ADIPOGENESIS	Details ...	197	-0.2361732	-1.0680834	0.30717185	0.3783189	1	2588	tags=19%, list=13%, signal=22%
HALLMARK_ SPERMATOGENESIS	HALLMARK_ SPERMATOGENESIS	Details ...	132	-0.14034766	-0.61015123	0.9958506	0.9962178	1	5174	tags=23%, list=26%, signal=31%
